# The effect of BI 409306 on heart rate in healthy volunteers: a randomised, double-blind, placebo-controlled, crossover study

**DOI:** 10.1007/s00228-022-03274-6

**Published:** 2022-01-28

**Authors:** Fabian Müller, Michael Sand, Glen Wunderlich, Jasmin Link, Christian Schultheis, Chantaratsamon Dansirikul, Rucha Sane, Roman Laszlo, Jürgen M. Steinacker

**Affiliations:** 1 Boehringer Ingelheim Pharma GmbH & Co. KG, Biberach an der Riss, Germany; 2 Boehringer Ingelheim Pharmaceuticals, Inc, Ridgefield, CT USA; 3 Division of Sports and Rehabilitation Medicine, Ulm University Hospital, Ulm, Germany

**Keywords:** BI 409306, Phosphodiesterase 9, Cardiovascular safety, Cardiopulmonary exercise testing, Pharmacokinetics

## Abstract

**Purpose:**

The potent, selective phosphodiesterase-9A inhibitor BI 409306 may be beneficial for patients with attenuated psychosis syndrome and could prevent relapse in patients with schizophrenia. Transient BI 409306-dependent increases in heart rate (HR) demonstrated previously necessitated cardiac safety characterisation. We evaluated cardiac effects of BI 409306 in healthy volunteers during rest and exercise.

**Methods:**

In this double-blind, three-way crossover study, volunteers received placebo, BI 409306 50 mg or 200 mg in randomised order (same treatment on Days 1 [resting] and 3 [exercise]). Cardiopulmonary exercise testing was performed twice post treatment on Day 3 of each period. BI 409306-mediated effects on placebo-corrected change from baseline in resting HR (ΔΔHR) were evaluated based on exposure–response analysis and a random coefficient model. Adverse events (AEs) were recorded.

**Results:**

Overall, 19/20 volunteers completed. Resting ΔΔHR versus BI 409306 concentration yielded a slope of 0.0029 beats/min/nmol/L. At the geometric mean (gMean) maximum plasma concentration (*C*_max_) for BI 409306 50 and 200 mg, predicted mean (90% CI) ΔΔHRs were 0.80 (− 0.76, 2.36) and 5.46 (2.44, 8.49) beats/min, respectively. Maximum adjusted mean differences from placebo (90% CI) in resting HR for BI 409306 50 and 200 mg were 3.85 (0.73, 6.97) and 4.93 (1.69, 8.16) beats/min. Maximum differences from placebo in resting HR occurred at/near gMean *C*_max_ and returned to baseline after approximately 4 h. The proportion of volunteers with AEs increased with BI 409306 dose.

**Conclusion:**

Observed hemodynamic effects following BI 409306 administration were of low amplitude, transient, and followed the pharmacokinetic profile of BI 409306.

## Introduction

Attenuated psychosis syndrome (APS) and schizophrenia have been associated with abnormalities in glutamatergic neurotransmission related to impaired *N*-methyl-D-aspartic acid (NMDA) receptor signalling [1–3]. Activation of the NMDA receptor leads to postsynaptic signalling events via increases in second messengers such as cyclic guanosine monophosphate (cGMP) [4]. It has been suggested that during NMDA receptor hypofunction, inhibition of phosphodiesterase 9A (PDE9A), which hydrolyses cGMP, could increase intracellular cGMP concentrations, thus restoring NMDA receptor signalling. This restoration of function would lead to strengthened synaptic plasticity and stabilisation via enhanced long-term potentiation, and could therefore benefit patients with APS and schizophrenia relapse [5–7].

BI 409306, a potent and selective PDE9A inhibitor that may improve NMDA signalling, is currently under clinical development as a potential treatment for APS [8] and for prevention of relapse in schizophrenia [9]. Clinical studies have shown that single and multiple doses of BI 409306 are well tolerated, and no cardiovascular safety signals were observed in healthy volunteers [7, 10–13] or in patients with schizophrenia [14]. However, there were some concerns about potential cardiovascular effects. In one study, one healthy volunteer in the 350 mg dose group experienced an episode of transient sinus tachycardia, which was of mild intensity and did not exceed 120 beats/min [7]. In another Phase I study, a brief and transient increase in supine heart rate (HR) was observed in volunteers with predicted cytochrome P450 isoform 2C19 (CYP2C19) poor metaboliser status who received a single dose of BI 409306 100 mg [15]. Based on retrospective pharmacometric modelling of the available combined Phase I data (unpublished data), an increase in HR of 7–13 beats/min was projected at high plasma exposure (i.e. at the 95th percentile of the observed maximum plasma concentration in ‘CYP2C19 poor metabolisers’ following a single dose of BI 409306 100 mg).

Considering these previous data regarding the potential cardiac effects of BI 409306, a dedicated safety evaluation with an emphasis on characterising cardiac safety was deemed important. Thus, the current trial was conducted in young, healthy volunteers with the anticipated therapeutic dose and a supra-therapeutic dose of BI 409306. The potential effects of BI 409306 on cardiopulmonary function were also assessed under exercise conditions. Specifically, the effects of BI 409306 on HR, oxygen uptake, and oxygen pulse are explored in this article.

## Methods

### Study design

**Fig. 1:**
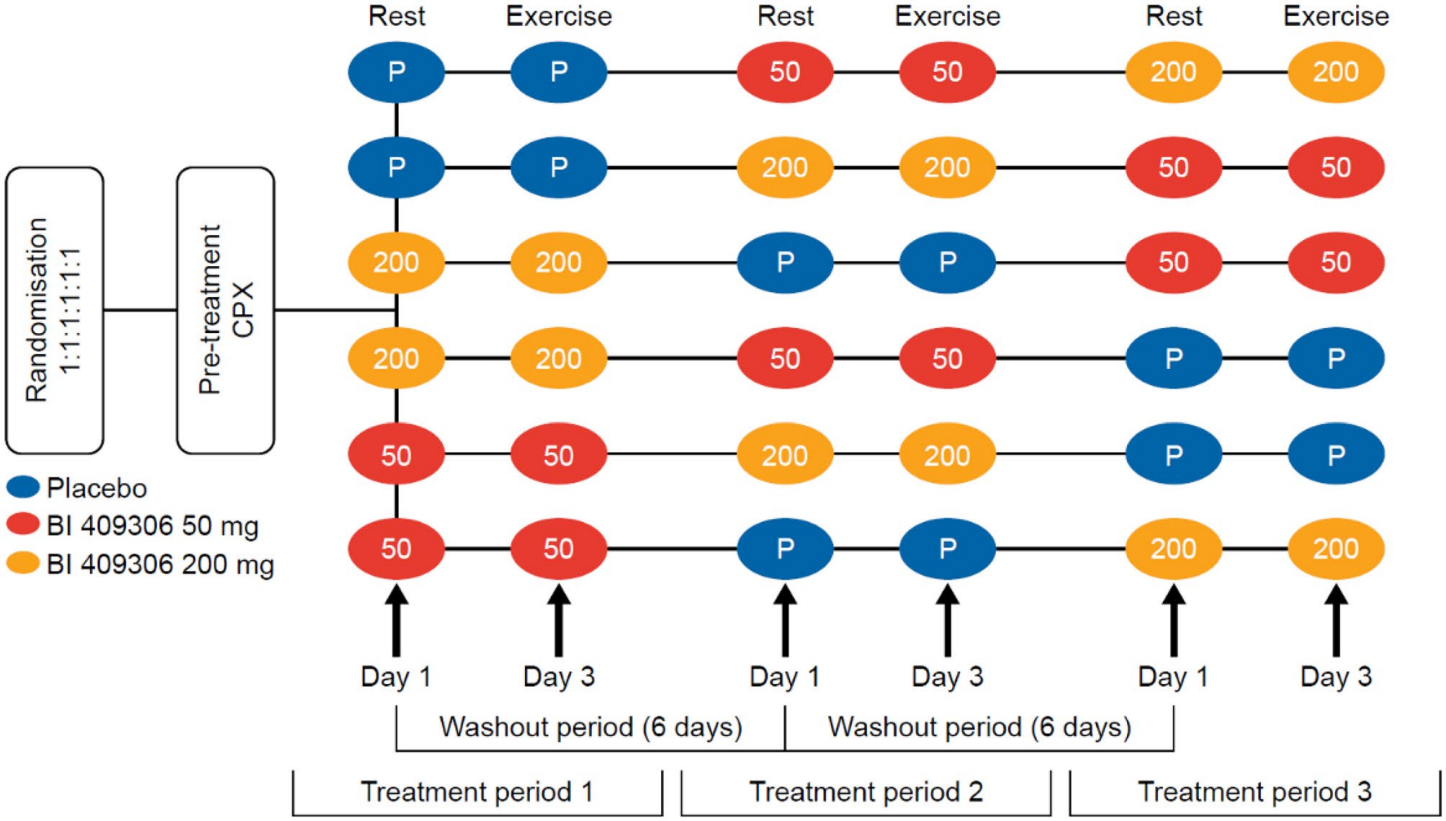
Study design. CPX cardiopulmonary exercise testing

This was a randomised, placebo-controlled, double-blind, double-dummy, three-way crossover study in healthy male volunteers (Fig. 1; clinicaltrials.gov: NCT02438683). Two doses of BI 409306 were used: 50 mg (anticipated therapeutic dose) and 200 mg (anticipated supra-therapeutic dose). The supra-therapeutic dose was chosen to allow for evaluation of potential cardiac effects at the upper level of the exposure range, thereby providing information that will allow the assessment of an acceptable safety margin. In a first-in-human trial, single doses of BI 409306 350 mg showed an acceptable safety and tolerability profile in young, healthy males genotyped as extensive metabolisers of CYP2C19, an enzyme involved in oxidative metabolism of BI 409306 [7]. As no volunteers with poor CYP2C19 metabolism were included in the present study, a lower dose of 200 mg was expected to be well tolerated, and was selected for use in the current trial.

Volunteers were randomised (1:1:1:1:1:1) to one of the 6 treatment sequences, in which they received placebo or BI 409306 (50 mg or 200 mg) at rest (Day 1) followed by the same treatment during exercise (Day 3). Each treatment sequence received the three treatments in a different order, as shown in Fig. 1.

Investigators and volunteers were blinded to trial treatments. BI 409306 was administered as 50-mg film-coated tablets, with each dose consisting of either 1 tablet of BI 409306 and 4 tablets of placebo (BI 409306 50 mg treatment group), 4 tablets of BI 409306 and 1 tablet of placebo (BI 409306 200 mg treatment group), or 5 tablets of placebo (placebo treatment group), to ensure that blinding was maintained.

Volunteers were admitted to the trial site in the morning before the first administration of trial medication in each period (Day 1) and in the evening before the second administration of trial medication (Day 2). Volunteers were then kept under medical surveillance for at least 10 h following administration of trial medication. During in-house stay, food and water intake was standardised and restricted to those provided by site staff.

For the evaluation of the electrocardiogram (ECG) endpoints, continuous ECGs were collected using 12-lead Holter recorders (CM 3000–12, GETEMED AG, Berlin, Germany) on Days 1 and 3 of each treatment period. On Day 1, triplicate ECGs were extracted under resting conditions. On Day 3, Holter ECGs were used to monitor and evaluate HR under exercise conditions. Immediately after the period for ECG extraction, plasma samples were taken for pharmacokinetic analysis. Holter ECGs were evaluated by a central ECG laboratory (nabios GmbH, Munich, Germany).

On rest (Day 1) and exercise (Day 3) days, plasma samples were collected before administration of the study medication and post administration at 20 min, 30 min, 45 min, then every 30 min from 1 to 3 h, and at 4 h, 6 h, and 10 h. On Day 1, one additional sample was collected at 8 h. On Day 3, two additional samples were collected at 2 h 20 min and 2 h 45 min after administration — this enabled identical relative blood sampling times during the two cardiopulmonary exercise (CPX) tests (see below) on that day. Samples were withdrawn using an indwelling venous catheter, or by venepuncture. BI 409306 plasma concentrations were measured using a validated liquid chromatography coupled to tandem mass spectrometry (LC–MS/MS) assay with [^13^C_2_, D_4_]BI 409603 as the internal standard. Chromatography was performed on an analytical reverse-phase column with gradient elution. Detection and quantification used electrospray ionisation in the positive ion mode. The lower limit of quantification was 1.50 nmol/L.

Prior to the first treatment period, two sessions (separated by at least 1 day) of CPX were performed (Ergostik, Geratherm Respiratory GmbH, Germany; CardioPart 12, AMEDTEC Medizintechnik GmbH, Germany, and ergometer ergoselect 200 BP, ergoline GmbH, Germany). For both sessions, CPX was performed using a ramp protocol: after 3 min of unloaded pedalling, workload was increased from 6 watts (W) by 25 W/min until maximal exertion. The purpose of the first CPX session was purely to familiarise the subject with the experimental setup; hence, the obtained data were not analysed further. The second CPX session was performed to obtain the workload (in W) at the individual ventilatory threshold (VT1). This workload, at approximately 50% of maximal oxygen consumption, allows continuous exercise without excess ventilation and without progressively increasing blood lactate [16, 17]. It is known that a steady-state HR is reached 3–5 min after the onset of submaximal exercise below the anaerobic threshold [18], allowing detection of BI 409306-induced changes in HR and other parameters of interest. This submaximal workload was then used during on-treatment exercise as described below. VT1 was determined manually by trained healthcare professionals based on previously published standard approaches [17].

On rest days (Day 1), volunteers were kept at rest for 4 h after drug intake. On exercise days (Day 3), volunteers performed two CPX sessions at 20 min after drug intake (close to expected geometric mean [gMean] maximum plasma concentration [*C*_max_]), and at 2 h 20 min after drug intake. Each CPX consisted of 3 min of low workload pedalling (‘warm-up’ at one-third of each subject’s individual VT1), 10 min of pedalling with constant, submaximal workload (at each subject’s individual VT1), and a 5-min recovery period without pedalling. Oxygen uptake and oxygen pulse, the latter as a surrogate for left ventricular stroke volume, were also analysed as parameters of further interest.

### Volunteers

Volunteers were healthy, non-smoking males aged 18–45 years with a BMI of 18.5–29.9 kg/m^2^, a waist-to-height ratio of < 0.5, and genetically determined not to be CYP2C19 poor metabolisers. Waist-to-height ratio was chosen in addition to BMI to better account for central obesity and ensure a homogenous trial population [19]. In principle, no concomitant therapy was allowed. In case concomitant therapy was necessary (e.g. for the treatment of adverse events [AEs]), the use of CYP2C19 inhibitors, PDE inhibitors, and CYP2C19 inducers were nevertheless to be avoided throughout the whole course of the trial.

Volunteers with repeated systolic blood pressure < 90 mm Hg or > 140 mm Hg, diastolic blood pressure < 50 mm Hg or > 90 mm Hg, or pulse rate < 45 beats/min or > 90 beats/min during screening were excluded, as were those with marked baseline prolongation of QT/QTc interval or any other relevant ECG finding at screening.

### Endpoints

The present report focuses on key endpoints related to HR, oxygen pulse, and oxygen uptake. Primary endpoints were the slope of the placebo-corrected change from baseline in resting HR (ΔΔHR) versus BI 409306 plasma concentration, 0–10 h after intake of trial medication (a positive slope [*β* > 0] indicates an increase in resting HR with increasing plasma concentrations), and the maximum difference (vs placebo) in change from baseline resting HR for each dose of BI 409306, 0–4 h after intake of trial medication.

Secondary endpoints included the slope of the placebo-corrected maximum HR during exercise versus plasma concentration of BI 409306, and the slope of the placebo-corrected difference between maximum HR during exercise and recovery HR (1 and 5 min after end of exercise) versus plasma concentration of BI 409306. Further endpoints included the slope of the placebo-corrected oxygen uptake and oxygen pulse (volume of oxygen consumed by the body per heartbeat) during exercise steady state versus plasma concentration of BI 409306.

### Pharmacokinetic outcome measures

Pharmacokinetic parameters of interest included *C*_max_, the time from dosing to maximum measured concentration of BI 409306 in plasma after single dose (*t*_max_), the area under the concentration–time curve (AUC) of BI 409306 in plasma over the time interval from 0 extrapolated to infinity after single dose (AUC_0 − ∞_), and the terminal half-life of BI 409306 in plasma (*t*_1/2_).

### Further safety and tolerability assessments

Safety and tolerability were assessed based on AE questioning, physical examination, safety laboratory tests, safety ECGs, vital signs (pulse rate, blood pressure), and suicidality (based on the Columbia Suicide Severity Rating Scale [C-SSRS]). Clinically relevant findings in any of these investigations, or in the Holter ECGs, were reported as AEs. Any suicidal ideation during the trial was also reported as an AE. Suicidal ideation with intention to act or with a specific plan and intent, or any suicidal behaviour during the trial, was reported as an SAE. Hepatic injury was defined as AE of special interest (AESI). The investigator assessed all AEs for causal relationship to trial drug treatment. AEs occurring within the residual effect period of BI 409306 (within 24 h after drug administration) were classified as on-treatment (i.e. treatment-emergent) AEs. AEs were coded using the Medical Dictionary for Regulatory Activities (MedDRA) version 18.0 and analysed according to the concept of volunteers with treatment-emergent AEs.

### Statistical analysis

All volunteers who received ≥ 1 dose of study drug were included in the treated set (TS), which was used for safety analyses (with the exception of ECG safety assessments). Those in the TS who provided ≥ 1 evaluable plasma concentration of BI 409306 (not affected by protocol violations relevant to the statistical evaluation of pharmacokinetic parameters) were included in the pharmacokinetic set (PKS), which was used for the descriptive analysis of pharmacokinetic concentrations and parameters. All volunteers in the TS who had ≥ 1 baseline, and ≥ 1 post-baseline assessment in 1 treatment period for ≥ 1 ECG interval endpoint were included in the ECG set (ECGS), which was used for all ECG analyses except those assessing the relationship between BI 409306 plasma concentration and ECG variables. Those in the ECGS who provided ≥ 1 valid drug plasma concentration and a corresponding ECG variable or oxygen variable in any period were included in the pharmacokinetic ECG set (PKECGS), which was used for exposure–response analyses.

The relationship between plasma concentrations and corresponding response variables was investigated using a random coefficient model including an overall fixed slope effect, together with a two-sided 95% confidence interval (CI) based on the *t*-distribution, as well as a random intercept and slope effect per subject (exposure response analysis, see also Garnett et al. [20]). Additionally, mean ΔΔHR and its 90% CI at the gMean of *C*_max_ for each active treatment (BI 409306 50 mg or 200 mg) were estimated. The maximum mean difference between BI 409306 treatment and placebo for the change from baseline in corresponding response variables was assessed using mixed-effect model repeat measurement, including period baseline, subject baseline, treatment, time, period, baseline × time interaction, treatment × time interaction, and period × time interaction as fixed effects.

## Results

### Study population

Overall, 20 volunteers entered and 19 completed the study. One subject in treatment sequence placebo/BI 409306 50 mg/BI 409306 200 mg discontinued trial participation due to AEs after the first dose of the second treatment period. All 20 volunteers were included in the TS and PKS, while 19/20 volunteers were included in the ECGS and the PKECGS. There were no relevant demographic imbalances between the volunteers entered in the different treatment sequences. Overall, the mean (SD) age and BMI of volunteers were 32.1 (8.4) years and 24.84 (2.56) kg/m2, respectively. Nineteen volunteers (95.0%) were Caucasian, and 1 subject (5.0%) was black/African American.

### Pharmacokinetics

**Fig. 2:**
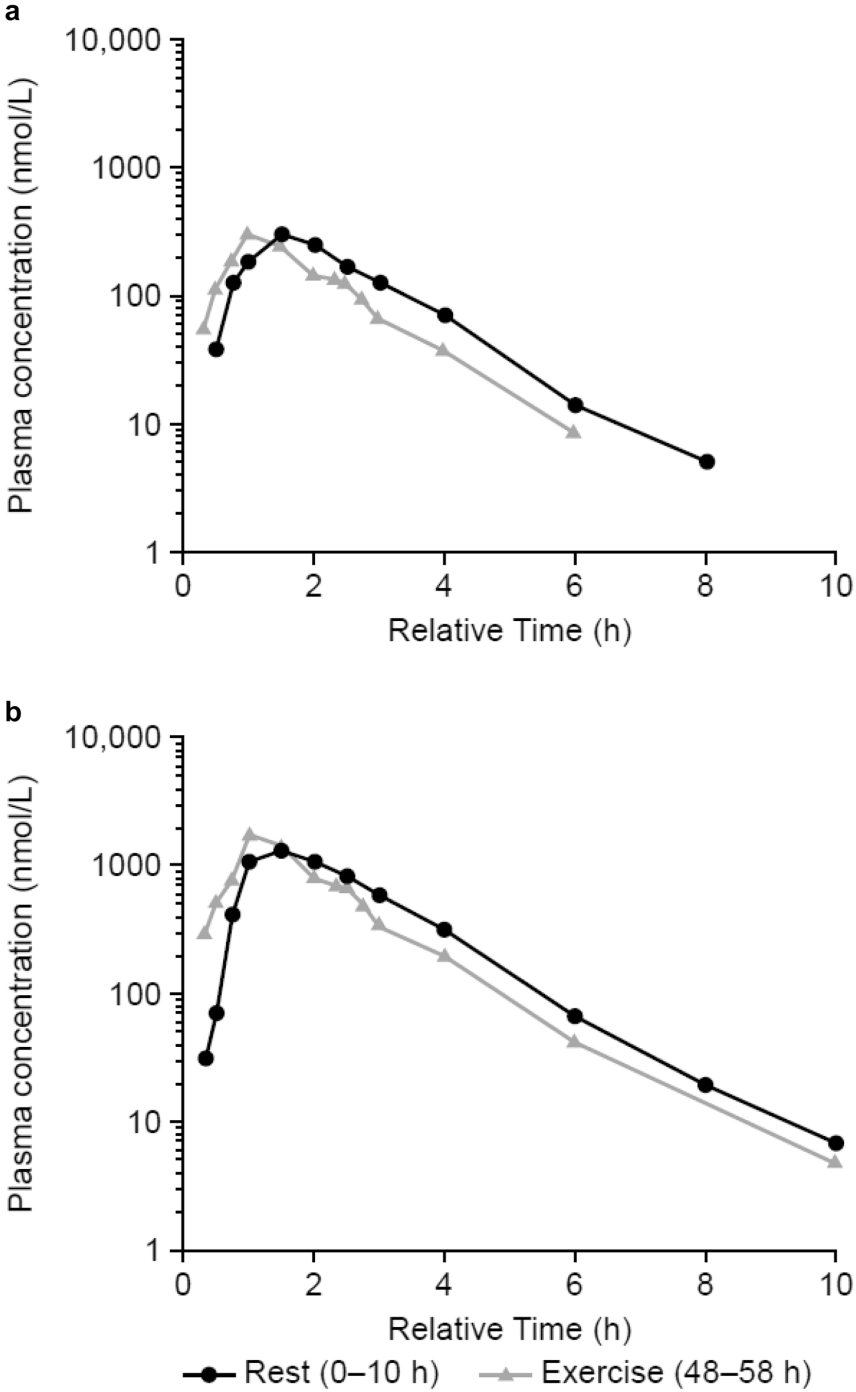
Geometric mean drug plasma concentration–time profiles of **a** BI 409306 50 mg or **b** BI 409306 200 mg under resting conditions (0–10 h) or under exercise conditions (48–58 h; PKECGS). Treatment was administered at 0 h on Day 1 (Rest) and at 48 h on Day 3 (Exercise). Relative time (*x*-axis) denotes time after drug administration on the respective treatment day. PKECGS pharmacokinetic electrocardiogram set

Pharmacokinetic parameters of BI 409306 (50 and 200 mg) under resting and exercise conditions are shown in Table 1. The exposures under both conditions were generally similar (Fig. 2). A very brief, transient plateau in BI 409306 plasma concentrations in the elimination phase was observed during the second CPX at both doses in individual volunteers (approximately 2 h 20 min post dose). This may not have been as evident during the first CPX as BI 409306 plasma concentrations were still increasing at that timepoint.

**Table 1:** Summary of pharmacokinetic parameters of BI 409306 under resting and exercise conditions

gMean (%gCV)	**BI 409306 50 mg**		**BI 409306 200 mg**	
	**Rest**	**Exercise**	**Rest**	**Exercise**
**C**_**max**_**, nmol/L**	433 (55.0)	388 (52.7)	2040 (72.5)	2210 (40.7)
**AUC**_**0 − ∞**_**, nmol∙h/L**	811 (59.4)	630 (60.4)	3790 (69.1)	3420 (52.2)
***t***_**1/2**_**, h**	1.03 (18.4)	0.985 (18.2)	1.17 (15.2)	1.11 (9.79)
***t***_**max**_**, h**^**a**^	1.5 (0.333–2.02)	1.0 (0.300–1.50)	1.0 (0.333–2.50)	1.0 (0.467–1.50)

*%gCV* geometric coefficient of variation, *AUC*_*0-∞*_ the area under the concentration–time curve of BI 409306 in plasma over the time interval from 0 extrapolated to infinity after single dose, *C*_*max*_ maximum plasma concentration, *gMean* geometric mean, *t*_*1/2*_ terminal half-life of BI 409306 in plasma, *t*_*max*_ time from dosing to maximum measured concentration of BI 409306 in plasma after single dose

^a^Median (range)

### Exposure–response analysis of resting ΔΔHR

**Fig. 3:**
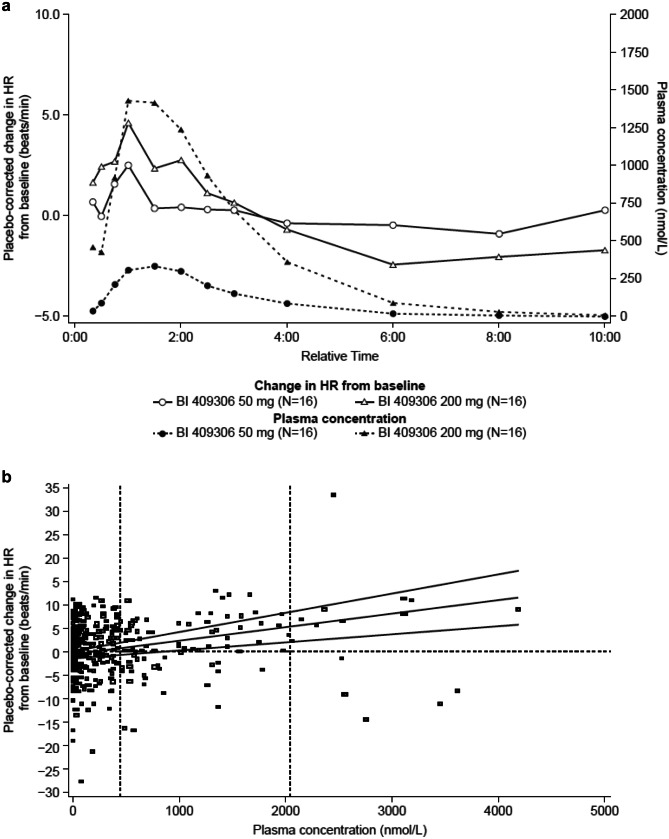
**a** Time profile of mean BI 409306 plasma concentrations and mean resting ΔΔHR and **b** relationship between BI 409306 plasma concentration and resting ΔΔHR (PKECGS). Panel **a** Solid lines represent placebo-corrected change in HR from baseline; dashed lines represent plasma concentration. Panel **b** Predicted values and 90% CI are represented by solid lines; gMean *C*_max_ at each dose level is represented by vertical lines. *C*_max_ maximum plasma concentration, gMean geometric mean, ΔΔHR, placebo-corrected change from baseline in resting heart rate, PKECGS pharmacokinetic electrocardiogram set

Maximum changes in resting HR occurred at/near the time of *C*_max_, approximately 1 h after administration of the study medication, and returned to baseline relatively rapidly, after approximately 4 h (Fig. 3A). Thus, no delayed effect of BI 409306 on HR was observed. The evaluation of the plasma concentration effect on ECG via the exposure–response model (linear relationship of ΔΔHR vs BI 409306 plasma concentration) yielded a slope of 0.0029 beats/min/nmol/L (95% CI: 0.0012, 0.0046). At the gMean C_max_ for BI 409306 50 mg, the predicted mean ΔΔHR was 0.80 beats/min (90% CI: − 0.76, 2.36); at the gMean C_max_ for the 200-mg dose, the predicted mean ΔΔHR was 5.46 beats/min (90% CI: 2.44, 8.49) (Fig. 3B).

### Repeated measures analysis of resting HR

The dose effect evaluation on ECG yielded a maximum adjusted mean change in resting HR of 3.85 beats/min (90% CI: 0.73, 6.97) and 4.93 beats/min (90% CI: 1.69, 8.16) for BI 409306 50 and 200 mg, respectively, which occurred at/near the time of *C*_max_ for each dose.

### Exposure–response analysis of placebo-corrected maximum HR during exercise and recovery

**Fig. 4:**
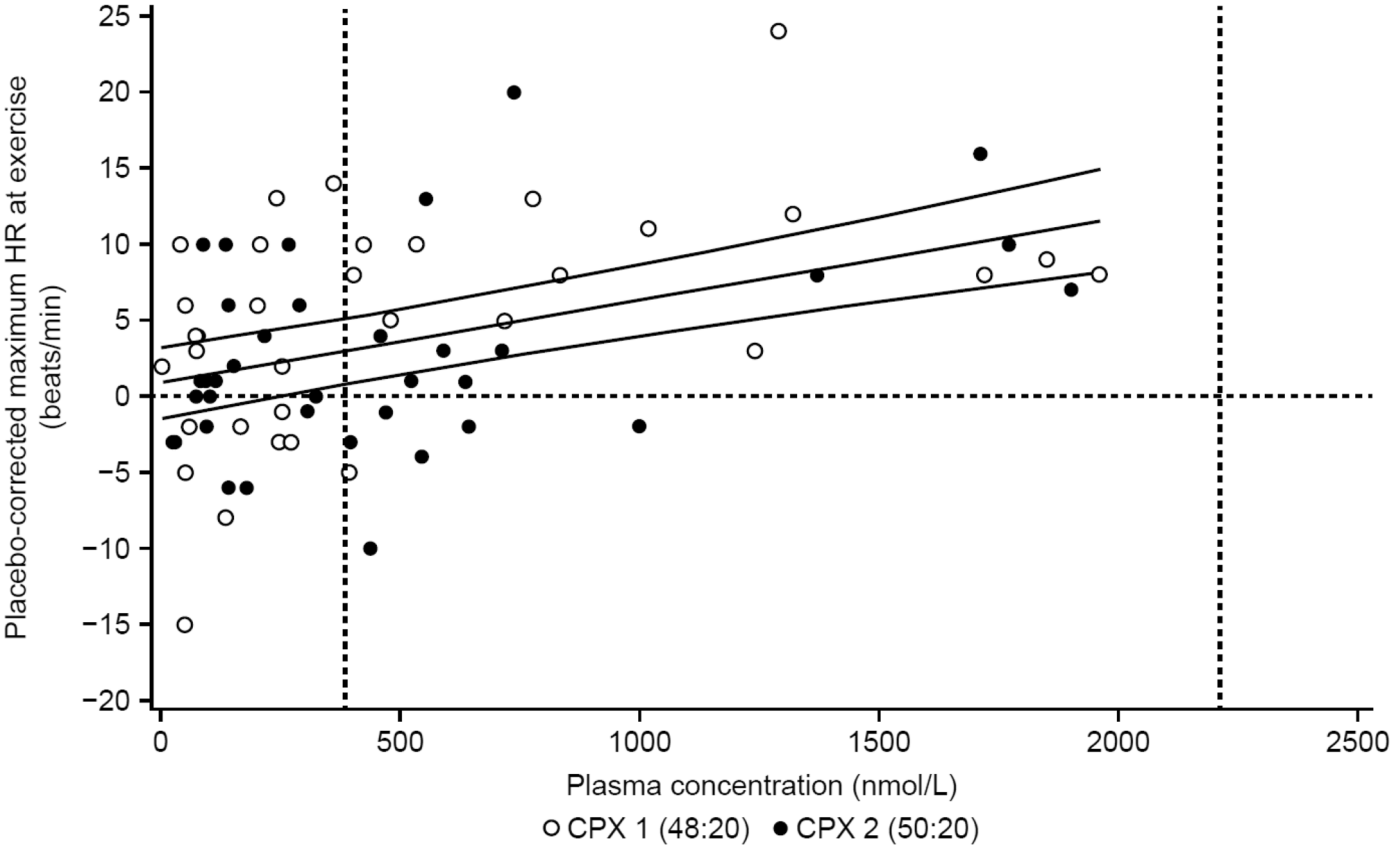
Relationship between placebo-corrected maximum HR under exercise conditions and BI 409306 plasma concentration (PKECGS). Predicted values and 90% CI are shown by solid lines; gMean *C*_max_ at each dose level is shown by vertical lines. *C*_max_ gMean maximum plasma concentration, CPX cardiopulmonary exercise testing, HR heart rate, gMean geometric mean, PKECGS pharmacokinetic electrocardiogram set

Assessment of placebo-corrected maximum HR during exercise versus BI 409306 plasma concentration yielded a slope of 0.0054 beats/min/nmol/L (95% CI: 0.0032, 0.0076). At the gMean C_max_ for BI 409306 50 mg, the predicted mean increase in placebo-corrected maximum HR was 2.97 beats/min (90% CI: 0.80, 5.14). Predicted mean placebo-corrected maximum HR was not extrapolated to gMean *C*_max_ for the 200 mg dose during exercise because all volunteers completed exercise before *C*_max_ was reached; therefore, maximum HR during exercise did not coincide with *C*_max_. The effect of BI 409306 on placebo-corrected maximum HR at the maximum individual plasma concentration during exercise (1960 nmol/L) is therefore shown, as opposed to the effect at *C*_max_. During exercise, the predicted mean placebo-corrected maximum increase in HR was 11.51 beats/min (90% CI: 8.14, 14.88; post hoc analysis; Fig. 4).

The HR decrease after the end of exercise was more pronounced at higher BI 409306 plasma concentrations; at the gMean *C*_max_ for BI 409306 50 mg and 200 mg, the predicted placebo-corrected difference between maximum HR during exercise and after 5 min recovery was 0.58 beats/min (90% CI: − 1.07, 2.23) and − 4.79 beats/min (90% CI: − 8.21, − 1.37), respectively. Overall, during recovery (1–5 min after cessation of exercise), HR decreased and there was no delay in recovery as a result of BI 409306 exposure.

### Exposure–response analysis of placebo-corrected oxygen uptake and oxygen pulse during exercise

**Fig. 5:**
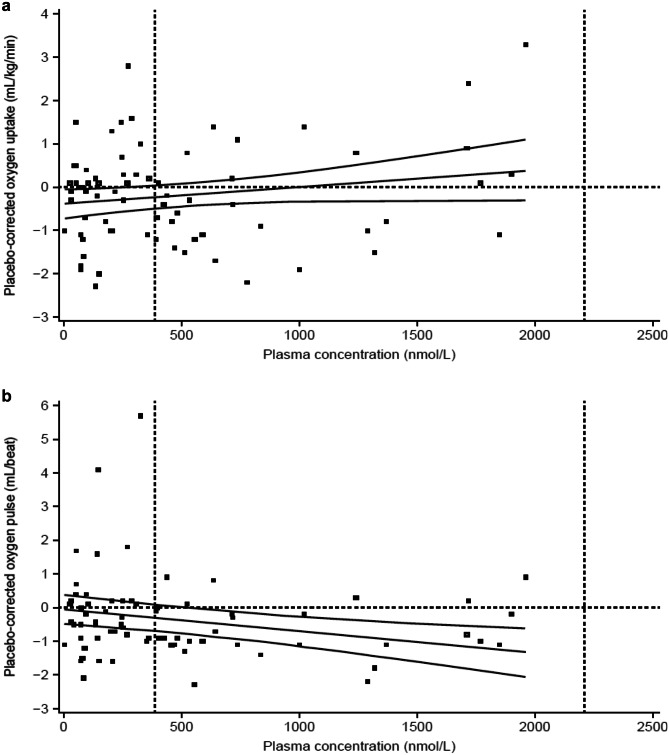
Relationship between plasma concentrations of BI 409306 and **a** placebo-corrected oxygen uptake and **b** placebo-corrected oxygen pulse under exercise conditions (PKECGS). Predicted values and 90% CI are shown by solid lines; gMean *C*_max_ at each dose level is shown by vertical lines. *C*_max_ gMean maximum plasma concentration, gMean geometric mean, PKECGS pharmacokinetic electrocardiogram set

Assessment of the relationship between placebo-corrected oxygen uptake during exercise versus plasma concentration of BI 409306 provided a slope of 0.0004 mL/kg/min/(nmol/L) (95% CI: − 0.0001, 0.0009). At the gMean *C*_max_ for BI 409306 50 mg, the predicted placebo-corrected oxygen uptake during exercise was − 0.23 mL/kg/min (90% CI: − 0.49, 0.04). It appeared that BI 409306 at the plasma concentrations achieved in the trial did not have a clinically relevant impact on oxygen uptake (Fig. 5A).

Assessment of the relationship between placebo-corrected oxygen pulse during exercise versus plasma concentration of BI 409306 provided a slope of − 0.0007 mL/beat/(nmol/L) (95% CI: − 0.0012, − 0.0002). At the gMean *C*_max_, the predicted placebo-corrected oxygen pulse during exercise was − 0.30 mL/beat (90% CI: − 0.69, 0.10) for BI 409306 50 mg, demonstrating a minor BI 409306 dose-dependent decrease in oxygen pulse (Fig. 5B).

### Adverse events

Treatment-emergent AEs were reported for 13/20 (65.0%) volunteers (Table 2). The most frequently reported AEs by system organ class were eye disorders (9/20 [45.0%] volunteers) and nervous system disorders (7/20 [35.0%] volunteers); none of the AEs in these categories were reported in volunteers receiving BI 409306 50 mg. AEs increased with dose but with generally similar rates at rest and during exercise. One subject discontinued treatment after experiencing mild ventricular extrasystoles, mild palpitations, and mildly increased HR following BI 409306 50 mg. All three AEs resolved without therapeutic intervention after 1 h. There were no clinically relevant, treatment-emergent findings in safety-related laboratory assessments, and the C-SSRS assessment revealed no suicidal ideation or behaviour. No AEs of severe intensity, AESIs, or SAEs were reported.

**Table 2:** Summary of volunteers with treatment-emergent adverse events by treatment, primary system organ class, and preferred term (TS)

	**Placebo**		**BI 409306 50 mg**		**BI 409306 200 mg**		**Total BI 409306**^**a**^ ***N* = 20**	**Total** ***N* = 20**
	**Rest** ***N* = 20**	**Exercise** ***N* = 20**	**Rest** ***N* = 20**	**Exercise** ***N* = 19**	**Rest** ***N* = 19**	**Exercise** ***N* = 19**		
**Any adverse event, *n* (%)**	6 (30.0)	2 (10.0)	3 (15.0)	1 (5.3)	9 (47.4)	7 (36.8)	12 (60.0)	13 (65.0)
** Drug-related adverse event, *n* (%)**	4 (20.0)	1 (5.0)	2 (10.0)	1 (5.3)	9 (47.4)	7 (36.8)	11 (55.0)	12 (60.0)
**Eye disorder**	1 (5.0)	0	0	0	7 (36.8)	6 (31.6)	8 (40.0)	9 (45.0)
** Photophobia**	0	0	0	0	3 (15.8)	2 (10.5)	3 (15.0)	3 (15.0)
** Visual brightness**	0	0	0	0	3 (15.8)	1 (5.3)	3 (15.0)	3 (15.0)
** Chromatopsia**	0	0	0	0	1 (5.3)	1 (5.3)	2 (10.0)	2 (10.0)
** Photopsia**	0	0	0	0	1 (5.3)	2 (10.5)	2 (10.0)	2 (10.0)
** Visual impairment**	1 (5.0)	0	0	0	0	0	0	1 (5.0)
**Nervous system disorder**	2 (10.0)	1 (5.0)	0	0	6 (31.6)	1 (5.3)	6 (30.0)	7 (35.0)
** Headache**	2 (10.0)	1 (5.0)	0	0	3 (15.8)	0	3 (15.0)	5 (25.0)
** Parosmia**	0	0	0	0	3 (15.8)	1 (5.3)	3 (15.0)	3 (15.0)
** Paresthesia**	0	0	0	0	1 (5.3)	0	1 (5.0)	1 (5.0)
**General disorder/administration site condition**	2 (10.0)	0	0	0	0	0	0	2 (10.0)
** Fatigue**	2 (10.0)	0	0	0	0	0	0	2 (10.0)
**Respiratory/thoracic/mediastinal disorder**	0	0	0	0	1 (5.3)	1 (5.3)	2 (10.0)	2 (10.0)
** Nasal congestion**	0	0	0	0	1 (5.3)	1 (5.3)	2 (10.0)	2 (10.0)
**Cardiac disorder**	0	0	1 (5.0)	0	0	0	1 (5.0)	1 (5.0)
** Palpitations**	0	0	1 (5.0)	0	0	0	1 (5.0)	1 (5.0)
** Ventricular extrasystoles**	0	0	1 (5.0)	0	0	0	1 (5.0)	1 (5.0)
**Infections/infestations**	0	0	1 (5.0)	0	0	0	1 (5.0)	1 (5.0)
** Oral herpes**	0	0	1 (5.0)	0	0	0	1 (5.0)	1 (5.0)
**Injury/poisoning/procedural complications**	0	1 (5.0)	0	0	0	0	0	1 (5.0)
** Muscle strain**	0	1 (5.0)	0	0	0	0	0	1 (5.0)
**Investigations**	0	0	1 (5.0)	0	0	0	1 (5.0)	1 (5.0)
** HR increase**	0	0	1 (5.0)	0	0	0	1 (5.0)	1 (5.0)
**Musculoskeletal/connective tissue disorder**	1 (5.0)	0	1 (5.0)	0	0	0	1 (5.0)	2 (10.0)
** Arthralgia**	1 (5.0)	0	0	0	0	0	0	1 (5.0)
** Extremity pain**	0	0	1 (5.0)	0	0	0	1 (5.0)	1 (5.0)
**Renal/urinary disorder**	0	0	0	0	1 (5.3)	0	1 (5.0)	1 (5.0)
** Pollakiuria**	0	0	0	0	1 (5.3)	0	1 (5.0)	1 (5.0)
**Skin/subcutaneous tissue disorder**	0	0	0	1 (5.3)	0	0	1 (5.0)	1 (5.0)
** Erythema**	0	0	0	1 (5.3)	0	0	1 (5.0)	1 (5.0)

^a^Volunteers with AEs during treatment with BI 409306 50 mg and/or during treatment with BI 409306 200 mg

## Discussion

BI 409306 concentration-dependent HR increases were observed under both resting and exercise conditions, with the maximum increase occurring at or near the time of *C*_max_. These increases in resting HR were transient, with a predicted mean of 5.46 beats/min at the expected supra-therapeutic dose. Furthermore, exercise testing did not suggest a clinically relevant impact on cardiac function. During recovery, HR decreased appropriately without delayed recovery. These findings are consistent with the brief, transient increase in HR observed in a previous study after a single dose of BI 409306 100 mg in CYP2C19 poor metabolisers [15]. The magnitude of the increase in HR was dependent on the plasma concentration of BI 409306; plasma concentrations in the present study were consistent with those reported previously [7].

CPX was chosen to evaluate a potential BI 409306-induced effect on HR under exercise conditions, as this method allows the determination of individual volunteers’ VT1 [18]. At fixed VT1-defined workloads below the anaerobic threshold, steady-state conditions are normally reached approximately 3–5 min after the onset of exercise and HR is subsequently maintained at reasonably constant levels. Using this VT1-defined workload allowed for the meaningful analysis of HR and other parameters of interest as they relate to BI 409306 administration.

Under steady state activity (such as in the current trial), oxygen uptake can be considered to be an indicator of oxygen consumption as a result of metabolic activity [17]. At the plasma concentrations achieved in this trial, BI 409306 did not have a relevant impact on oxygen uptake. As expected, due to BI 409306 concentration-dependent increases in HR, oxygen pulse as a CPX indicator of left ventricular stroke volume [17] showed a minor BI 409306 concentration-dependent decrease. Taken together, the hemodynamic effects of BI 409306 at therapeutic and supratherapeutic doses reported in this study were of low amplitude, transient, and followed the pharmacokinetic profile of BI 409306.

BI 409306 was well tolerated. Of note, the frequency of AEs was higher for the supratherapeutic 200 mg dose than for BI 409306 50 mg. The most common AEs for BI 409306 200 mg, which were those related to vision, may have occurred due to direct, transient retinal effects of BI 409306. This is supported by in vitro data linking PDE9A to processes within the cone pathway in the retina [21], and it is in line with results from previous trials of BI 409306 [7, 15], particularly one study in which vision-related AEs were only observed at a dose of 200 mg [10].

The mild palpitations, ventricular extrasystoles, and mild HR increase reported for a single subject during treatment with BI 409306 50 mg are events that can be observed in healthy volunteers [22, 23], and therefore, may not be directly linked to treatment. However, due to the timing of the onset of these symptoms (increased frequency of ventricular extrasystoles occurred shortly after BI 409306 50 mg intake), a causal relationship to the trial drug cannot be ruled out entirely. Nevertheless, these AEs were not considered clinically relevant overall.

One of the limitations of this study is the use of young and exclusively male, healthy volunteers. This population was selected because of the precision required for analysis of the ECG data, as sex- and age-related variability in ECG parameters could have precluded collection of interpretable data. In addition, the sample size was moderate, in line with the exploratory nature of the study. Strengths of this study are the highly controlled setting in a phase I unit, the robust crossover design, and the use of supratherapeutic and therapeutic doses of BI 409306 as well as placebo in a double-blind fashion. A further strength of this study is the novel evaluation of possible cardiac effects specifically under exercise conditions, which would not routinely be covered in a standard thorough QT trial monitoring potential cardiac effects of a candidate drug.

In conclusion, BI 409306 was well tolerated in young, healthy male volunteers. Transient BI 409306 concentration-dependent increases in HR of low amplitude were observed under resting and exercise conditions. These effects followed the pharmacokinetic profile of BI 409306, and analysis of HR recovery indicated no delay as a result of BI 409306. There was no effect of BI 409306 on oxygen uptake, and oxygen pulse showed a minor BI 409306 concentration-dependent decrease.
